# Digital Health Solutions to Control the COVID-19 Pandemic in Countries With High Disease Prevalence: Literature Review

**DOI:** 10.2196/19473

**Published:** 2021-03-10

**Authors:** Sharareh R Niakan Kalhori, Kambiz Bahaadinbeigy, Kolsoum Deldar, Marsa Gholamzadeh, Sadrieh Hajesmaeel-Gohari, Seyed Mohammad Ayyoubzadeh

**Affiliations:** 1 Department of Health Information Management Tehran University of Medical Sciences Tehran Iran; 2 Modeling in Health Research Center Institute for Future Studies in Health Kerman University of Medical Sciences Kerman Iran; 3 School of Paramedicine Shahroud University of Medical Sciences Shahroud Iran; 4 Medical Informatics Research Center Institute for Futures Studies in Health Kerman University of Medical Sciences Kerman Iran

**Keywords:** COVID-19, digital health, information technology, telemedicine, electronic health

## Abstract

**Background:**

COVID-19, the disease caused by the novel coronavirus SARS-CoV-2, has become a global pandemic, affecting most countries worldwide. Digital health information technologies can be applied in three aspects, namely digital patients, digital devices, and digital clinics, and could be useful in fighting the COVID-19 pandemic.

**Objective:**

Recent reviews have examined the role of digital health in controlling COVID-19 to identify the potential of digital health interventions to fight the disease. However, this study aims to review and analyze the digital technology that is being applied to control the COVID-19 pandemic in the 10 countries with the highest prevalence of the disease.

**Methods:**

For this review, the Google Scholar, PubMed, Web of Science, and Scopus databases were searched in August 2020 to retrieve publications from December 2019 to March 15, 2020. Furthermore, the Google search engine was used to identify additional applications of digital health for COVID-19 pandemic control.

**Results:**

We included 32 papers in this review that reported 37 digital health applications for COVID-19 control. The most common digital health projects to address COVID-19 were telemedicine visits (11/37, 30%). Digital learning packages for informing people about the disease, geographic information systems and quick response code applications for real-time case tracking, and cloud- or mobile-based systems for self-care and patient tracking were in the second rank of digital tool applications (all 7/37, 19%). The projects were deployed in various European countries and in the United States, Australia, and China.

**Conclusions:**

Considering the potential of available information technologies worldwide in the 21st century, particularly in developed countries, it appears that more digital health products with a higher level of intelligence capability remain to be applied for the management of pandemics and health-related crises.

## Introduction

The novel disease COVID-19, caused by the novel coronavirus SARS-CoV-2, was originally recognized in December 2019 as a case of pneumonia in Wuhan, China; it has since become a global pandemic, affecting most countries worldwide [1]. On March 11, the World Health Organization announced the outbreak of a pandemic and asked for coordinated mechanisms to support readiness and rapid response to the infection across the world's health sectors [2]. As the incidence of COVID-19 continues to rise, health care systems are rapidly facing growing clinical demands [3]. Operational management of a pandemic in the era of modern medicine requires novel technologies, such as digital health, that can support the management of COVID-19 cases in different stages [4]. Digital health as an application of information technology has already been used to improve health care organizations; for example, the National Health Service (NHS) in the United Kingdom has established the NHS Digital information center [5]. Digital health is defined as information technologies that can be applied in three aspects: digital patients, digital devices, and digital clinics. A digital patient is a patient who uses and engages with mobile health (mHealth) devices to change and sustain their behavior, including technologies such as telemedicine, patient self-measurements, and digital retention. Digital devices help solve clinical problems and include smartphone-connected rhythm monitoring devices, wireless and wearable devices, and implantable and ingestible sensors. The digital clinic aspect focuses on generating mHealth data, analyzing it so that it is clinically meaningful, and integrating it within clinical workflows. Aspects of digital clinics include precision-based mHealth and n-of-1 designs, population-based mHealth interventions in resource-limited areas, and mHealth regulation and integration [6].

During the COVID-19 pandemic, digital health–based tools may support organizations and societies more efficiently. They are useful for instant, widespread distribution of information, real-time transmission tracking, virtual venue creation for meetings and official day-to-day operations, and telemedicine visits for patients [7-12]. Such applications during the COVID-19 pandemic have been reported in several publications [13-15]. During the recent months of the COVID-19 outbreak, as countries and their responsible organizations such as health ministries and other officials have focused on controlling the pandemic, many supportive and reliable informatics infrastructures have been developed [12]. These infrastructures were applied in practice to prepare to manage an exponential increase in patients with COVID-19. Various digital health strategies have been used for disease control in different countries. A study conducted by Calton et al [16] provided some tips for applying telemedicine as a means to reduce the transmission of COVID-19. A study conducted by Moazzami et al [17] focused on employing telemedicine to prevent disease among health care providers. A study conducted by Keesara et al [18] referred to the capabilities and potential of digital health to fight COVID-19. However, they reviewed digital health–related solutions in general to address how this technology can support health care systems through introducing various strategic roles, such as surveillance, screening, triage, diagnosis, and monitoring, and contact tracing; no data regarding the use of this approach in practice for fighting COVID-19 were provided [19]. Fagherazzi et al [20] emphasized that the great potential of digital technology for COVID-19 control should be considered at the top level of health systems; they also discussed the challenges that policy makers may face in controlling the crisis using digital solutions. Furthermore, in a macro vision, they revealed the required societal and environmental restructuring required for successfully applying digital health technology to control COVID-19, including the health care system, government, public, industry, environment, and energy [15]. These reviews depict a general image regarding the requirement of digital system use and their applications worldwide [21], with no focus on any specific application in a specific country or region. Although these studies have shed light on the topic of applying digital health solutions for COVID-19 control, there is a gap of deep understanding regarding the application of these technologies in countries where COVID-19 is highly prevalent.

Therefore, this study aimed to review and analyze applied information technology and digital health–related strategies to control the COVID-19 pandemic in the 10 countries with the highest prevalence of the disease.

## Methods

In this review study, the databases of Google Scholar, PubMed, Web of Science, and Scopus were searched in August 2020 to retrieve publications from December 2019 to August 15, 2020. The combination of keywords for searching is shown below:

(“Corona virus” OR “COVID 19” OR “coronavirus”) AND (computer OR internet OR web* OR mobile OR smart OR email OR video confer* OR telecommunication OR ICT OR “information technology” OR ehealth OR telehealth OR mHealth OR telecare OR telehealth OR telemedicine OR telemonitoring OR digital OR wearable OR IoT OR cloud) AND (Italy OR Spain OR USA OR France OR UK OR Iran OR China OR Netherlands OR Germany OR Belgium)

The inclusion criteria were publications that introduce digital health applications to manage and control COVID-19 in humans, and the exclusion criteria were non-English publications, publications with no abstract, research on data analysis and modeling for prediction of epidemiological parameters, letters to the editor, and review studies. Data were analyzed using descriptive methods. Qualitative analysis of the included studies was performed based on predefined categories. A summary of the reviewed articles is provided in Table 1. Several items were analyzed in each paper, including (1) publication month; (2) country (Italy, Spain, United States, France, United Kingdom, Iran, China, the Netherlands, Germany, and Belgium, as they were the countries where COVID-19 was most prevalent according to the Worldometer website [22]); (3) purpose of the study, including screening, prevention, diagnosis, treatment, and follow-up of cases (defined as follows: screening: no symptom + no contact with COVID-19 patients; prevention: no symptom + contact with COVID-19 patients with no symptoms; diagnosis: having disease symptoms; treatment of COVID-19 cases: decreasing symptoms dramatically; and follow-up: discharged cases with the fewest symptoms); (4) scope and territory (village, city, region/province, state, country, and international), (5) digital tools, including robots, the Internet of Things, videoconferencing, web-based systems, cloud-based systems, wearable devices, clinical decision support systems (CDSSs), intelligent systems, smartphones, mobile apps, telecommunication systems, websites, digital media, and digital quick response (QR) codes.

**Table 1:** Details of the reviewed papers that discussed the application of digital health tools to control the COVID-19 pandemic.

Author	Journal	Publication month (2020)	Country	Purpose	Scope and territory	Applied digital tools	Application of digital tools
Kamel and Geraghty [23]	*International Journal of Health Geographics*	March	China	Prevention	International	Web-based systems, mobile apps, GIS^a^	Widespread distribution of information and real-time tracking of transmission
Yang et al [24]	*Clinical Oral Investigations*	May	China	Treatment and follow-up	Country	Web-based systems, mobile apps	Telemedicine visits for patients
Meng et al [25]	*International Journal of Clinical Pharmacy*	April	China	Treatment	Region	Cloud-based systems, smartphones, telecommunication systems	Provision of pharmaceutical care activities to patients and physicians by pharmacists
Ohannessian et al [26]	*JMIR Public Health and Surveillance*	February	France	Prevention	Country	Videoconferencing	Offering telemedicine visits for patients
Pan et al [27]	*Microbes and Infection*	February	China	Prevention	Country	Mobile apps	Widespread distribution of information and real-time tracking of transmission
Pan et al [28]	*Irish Journal of Medical Science*	March	China	Screening and prevention	City and country	Mobile apps	Real-time tracking of transmission
Sun et al [29]	*Annals of Intensive Care*	March	China	Treatment	State	Intelligent systems	Early warning systems and screening procedures for patients
Hernández-Garcia and Gimenez-Júlvez [30]	*JMIR Public Health and Surveillance*	April	Collaboration of the United States, Spain, Switzerland, the United Kingdom, Sweden, and Canada	Screening and prevention	International	Websites and digital media	Widespread distribution of information
Hua and Shaw [31]	*International Journal of Environmental Research and Public Health*	March	China	Screening, prevention, and follow-up	Region/ province	Web-based systems, smartphones, websites, digital media, digital QR^b^ codes	Widespread distribution of information, real-time tracking of transmission, provision of information about “fake news” and rumors
Drew et al [32]	*Science*	May	United Kingdom, United States	Screening	International	Mobile app	Widespread distribution of information, real-time tracking of transmission
Franco et al [33]	*Global Spine Journal*	June	United States	Treatment	State	Videoconferencing, telephone	Offering telemedicine visits for patients
Gilbert et al [34]	*BMJ Open Quality*	May	United Kingdom	Prevention	City	Videoconferencing, telephone	Offering telemedicine visits for patients
Giudice et al [35]	*International Journal of Environmental Research and Public Health*	May	Italy	Follow-up	Region	Videoconferencing	Offering telemedicine visits for patients
Gong et al [36]	*Journal of Medical Internet Research*	April	China	Prevention	Country	Telecommunication system	Offering telemedicine visits for patients
Gong et al [37]	*Journal of Medical Internet Research*	April	China	Screening	City	Cloud-based system, mobile app, CDSS^c^	Screening of cases and detection of patients
Goodman-Casanov et al [38]	*Journal of Medical Internet Research*	April	Spain	Prevention	Country	Telecommunication system	Widespread distribution of information, support for home care and patient self-care
Grange et al [39]	*Applied Clinical Informatics*	April	United States	Prevention, diagnosis, treatment, screening	State	Videoconferencing, CDSS, telecommunication	Offering telemedicine visits for patients
Grenda et al [40]	*Annals of Surgery*	August	United States	Diagnosis, treatment	City	Telecommunication, videoconferencing	Offering telemedicine visits for patients
Grossman et al [41]	*Neurology*	June	United States	Diagnosis, treatment	City	Smartphone, mobile apps	Offering telemedicine visits for patients
Hames et al [42]	*Journal of Psychotherapy Integration*	April	United States, Canada	Prevention	Country	Telecommunication system	Training
Hanna et al [43]	*Modern Pathology*	June	United States	Prevention, diagnosis	City	Telecommunication system	Diagnosis
Hom et al [44]	*Journal of Psychotherapy Integration*	April	United States	Prevention, treatment	City	Videoconferencing	Telemedicine visits for patients, training
Itamura et al [45]	*OTO Open*	April	United States	Prevention	Country	Videoconferencing	Telemedicine visits for patients
Judson et al [46]	*Journal of the American Medical Informatics Association*	June	United States	Prevention	State	Website	Screening of cases and detection of patients
Wu et al [47]	*European Respiratory Journal*	June	China, Italy, Belgium	Diagnosis	International	CDSS	Classification of patients in triage to find the best route
Wang et al [48]	*JMIR mHealth and uHealth*	June	China	Prevention	Country	Mobile app (WeChat)	Early tracing and quarantine of potential sources of infection
Timmers et al [49]	*JMIR mHealth and uHealth*	June	The Netherlands	Prevention	Country	Mobile app	Education, self-assessment, and symptom monitoring
Pepin et al [50]	*Journal of Medical Internet Research*	June	France	Prevention	International	Wearable devices and activity trackers	Definition of the level of quarantine
Rabuna et al [51]	*Telemedicine and e-Health*	June	Spain	Prevention	Rural area	TELEA digital web platform	Real-time tracking and monitoring of patients; follow-up of patients by telephone, videoconferencing, and email
Cheng et al [52]	Community Mental Health Journal	July	United States, Canada, Australia	Prevention	International	Mobile app	Peer-to-peer psychological support for Wuhan health care professionals at the front line of the crisis
Castaldi et al [53]	*Acta Biomedica*	July	Italy	Prevention	Region	Social media	Assessment of the dynamic burden of social anxiety through analysis of data from Facebook and Twitter
Blake et al [54]	*International Journal of Environmental Research and Public Health*	July	United Kingdom	Prevention	Country	Digital learning package using agile methodology	Provision of psychologically safe spaces for staff through providing a three-step e-package with evidence-based guidance

**^a^**GIS: geographic information system.

^b^QR: quick response.

^c^CDSS: clinical decision support system.

## Results

The search of scientific databases and manual searches retrieved 771 relevant articles. The titles and abstracts of all the retrieved publications were evaluated by two authors. Disagreements between the two evaluators were discussed and resolved by consensus. After removal of duplicates, 292 articles remained at this stage. Next, 260 publications were removed because they did not meet the inclusion criteria. Afterward, four authors independently reviewed the full text of the remaining publications (N=32). The reviewed papers were studied based on the variables shown in Table 1 and the different distributions discussed below.

For the purpose of this review, studies published from December 2019 to August 15, 2020, were reviewed. The survey identified 32 papers that demonstrated digital health applications to fight the COVID-19 pandemic. The distribution by publication month revealed that the publication of studies regarding digital health and COVID-19 began in February 2020, and the distribution of the 32 publications by month is February, 2 (6%); March, 5 (16%); April, 9 (28%); May, 3 (9%); June, 9 (28%); July, 3 (9%); and the first half of August, 1 (3%).

The projects of digital health application for COVID-19 control were deployed at different geographical levels, from international to rural. Six countries carried out six international projects, and the most common collaborations were among European countries, the United States, China, and Australia. The digital health projects at the international level mainly aimed to track real-time transmission and infected cases, define the level of quarantine, and enable peer-to-peer consultation to support care providers in other countries phytologically and scientifically. The studies of digital health projects for a given purpose in the 32 studies were most frequently conducted at the country level (n=10, 31%), and the other geographical levels were state (n=4, 13%), region (n=3, 9%), city (n=8, 25%), and rural (n=1, 3%). The United States was the country with the highest number of studies of digital health projects to fight COVID-19 (12/32, 38%), and these 12 studies varied the most in geographical scale, including international (n=3, 25%), state (n=3, 25%), country (n=2, 17%), and city (n=4, 13%) levels. The other studied countries ranked by the number of conducted studies were China (11/32, 34%); the United Kingdom (4/32, 13%); Canada, Spain, and Italy (3/32, 9%); Belgium and France (2/32, 6%); and the Netherlands (1/32, 3%).

To show the applied approaches of digital health for certain methods of COVID-19 control, the results were analyzed, and all the papers were categorized into six domains. These categories, their frequencies and percentages, and their applications for COVID-19 control are presented in Table 2. Some articles mentioned more than one approach to using digital health to control the COVID-19 pandemic.

**Table 2:** The frequency of digital health methods and their applications for COVID-19 pandemic control.

Domain number	Applied digital health solutions	COVID-19 control approaches	Digital health application projects (N=37), n (%)
1	Digital learning package, mobile apps, and web-based systems	Widespread distribution of information	7 (19)
2	GISs^a^, QR^b^ codes, and wearable devices	Real-time tracking of transmission, activity tracking, and quarantine-level analysis	7 (19)
3	Web-based systems and mobile apps, videoconferencing, and telephone	Telemedicine visit services and virtual venues for meetings	11 (30)
4	Cloud- and mobile-based systems	Self-care and patient monitoring, training, and diagnosis	7 (19)
5	Intelligent systems and CDSSs^c^	Early warning and detection, screening, and triage	4 (10)
6	Social media	Dynamic burden of the pandemic and analysis of its consequences	1 (3)

^a^GISs: geographic information systems.

^b^QR: quick response.

^c^CDSSs: clinical decision support systems.

According to the results, telemedicine visit services (11/37, 30%), especially in the United States (6/11, 54%), were the most commonly applied pandemic control approach. Using electronic methods to inform people about the disease, methods to prevent disease spread, and protection methods was the second-ranked approach, in addition to two other solutions of geographic information systems (GISs), QR codes, and wearable devices for real-time transmission tracking as well as cloud-based and mobile app usage for patient monitoring and self-care at home (all 7/37, 19%). A few studies were identified regarding the application of intelligent systems and CDSSs (4/37, 10%) and social media data analysis (1/37, 3%) for screening and burden of disease analysis purposes.

## Discussion

### Principal Findings

The COVID-19 pandemic has spread worldwide, costing lives and bringing upheaval and change to societies and economies. Although the global scientific community is racing to discover effective vaccines and therapeutics, the most essential defense remains public health measures such as personal hygiene and mass physical distancing. To successfully implement these two main measures, digital health and information technologies have emerged to support health systems, and they offer opportunities to reshape current health care systems. The aim of this study was to review the most significant digital health tools applied to fight COVID-19 in the 10 countries that have been most affected by the disease. These tools help governments and people to engage in strategies to control the COVID-19 pandemic through addressing the most urgent needs, including immediate outbreak response and impact mitigation. In China, which is the first country affected by the virus [55] and the most populous country globally, many researchers have worked on multiple aspects of SARS-CoV-2; it is the second most frequent origin country of the included studies. The burden of SARS-CoV-2 could be massive in populous countries; thus, these studies are worthy of investment in these countries. Studies that reported the development of models to predict epidemiological indicators were ignored, as they have not yet yielded any digital tools and require further development [56-59].

Distributing widespread information and tracking real-time transmission were the two most frequent goals of the studies. The former may originate from the importance of prevention in pandemic diseases as well as the simplest task of using information systems. The latter may be a focus in the literature because of the knowledge obtained from the previous experience of epidemics such as influenza and Zika virus [60-62]. Additionally, telemedicine visits for patients may be beneficial for populations because screening and follow-up of patients can be performed while maintaining social distancing in the population [63]. It appears that investigating the infrastructures needed for this technology could have great potential to mitigate these types of crises. In addition to the whole populations that can benefit from digital health technologies, more attention should be paid to interventions for travelers, as they can spread SARS-CoV-2 to other locations and even globally [64].

It has been shown that cell phones can be beneficial for health care [65]; due to their high influence among global populations, these tools are well suited for widespread distribution of information to these populations. Mobile apps are also used for tracking real-time transmission of SARS-CoV-2. Other potentially useful digital health tools are web-based apps and websites; these tools can also distribute information and track transmission. Videoconferencing and telecommunication also appear to be useful barriers to the spread of COVID-19 by enabling social distancing. Moreover, other industries may use teleservices to prevent the dissemination of disease.

Due to time limitations and the different times of onset of the epidemic in different countries, several digital health tools are not included in this paper. These tools have been reported in the news and other resources, and it may be valuable to discuss them as learned lessons for other countries fighting COVID-19. Therefore, we will review the digital health interventions in the different countries based on their available facilities and other requirements.

### China

In China, multiple approaches are being used to manage the COVID-19 pandemic, ranging from web-based and mobile-based systems to cloud-based systems, CDSSs, and intelligent systems. The total number of cases of COVID-19 in this country showed a slight increase after March 1, 2020, based on the data in [66]. However, this decrease in COVID-19 cases was affected by multiple factors, and the effect of eHealth tools on the decrease should be evaluated. China has widely applied an eHealth app named Health Code to indicate a person’s health status in the past day [67].

China has established a plan to spend approximately US $1.4 trillion on digital infrastructure. This infrastructure upgrade program includes developing 5G networks, industrial internet, data centers, and artificial intelligence [68], which could improve the country’s capability to fight pandemics.

### Italy and Spain

In contrast to Spain, Italy ranks among the four least advanced European countries in the Digital Economy and Society Index published by the European Commission [69], and approximately half the population of Italy has insufficient digital literacy [70]. The adoption of technology to prevent and manage the COVID-19 pandemic is unremarkable in these two countries. However, a coronavirus-tracking app was developed in Spain [71]. The statistics of total COVID-19 cases showed a dramatic increase after March 1, 2020, in both countries [66]. It appears that these countries should invest more in technologies to manage the pandemic.

### United States

The US government launched a portal [72] for the public that contains information on how to prevent and manage COVID-19. Moreover, the US Centers for Disease Control and Prevention website [73] contains more detailed medical information on the spreading mechanism, symptoms, prevention, and treatment of COVID-19.

### France and Belgium

The French app StopCovid was developed to trace infected people to control the spread of SARS-CoV-2. Privacy concerns arose regarding adoption of the app. Belgium has announced that a similar app adoption was canceled due to these issues [74].

### United Kingdom

The NHS in the United Kingdom works on nine main areas to digitally respond to the pandemic: provide digital channels for citizen guidance and triage; enable remote and collaborative care with systems and data; deliver digital services for NHS Test and Trace; identify and protect vulnerable citizens; support planning with data, analysis, and dashboards; get data and insights to research communities; support clinical trials; provide secure infrastructure and support additional capacity; and plan for recovery, restarting services, and new needs. The government has categorized initiatives in these areas [75].

### Iran

Although our study did not include any papers from this country, the Iranian Ministry of Health developed a national screening program website [76] to identify COVID-19 cases in the early stages.

### The Netherlands

The Netherlands is one of the leading countries in Europe in digital health care and data. Approximately 90% of the population has digital records, and the Dutch government has invested over 400 million euros (US $482,980,000) in digital health. Hospitals in the Netherlands have signed up for a COVID-19 web-based portal for sharing patient information. Video consultation was provided by more than 8000 health care providers [77].

### Germany

The Health Innovation Hub, established by Germany’s Ministry of Health, has published a list of trusted telemedicine applications. The services provided by these apps include remote consultation, risk assessment, and telemedicine services. Before 2018, the country did not allow remote consultations.

The German parliament passed the Digital Care Act, which acknowledged that digital health is crucial for fighting the COVID-19 pandemic [78].

**Figure 1:**
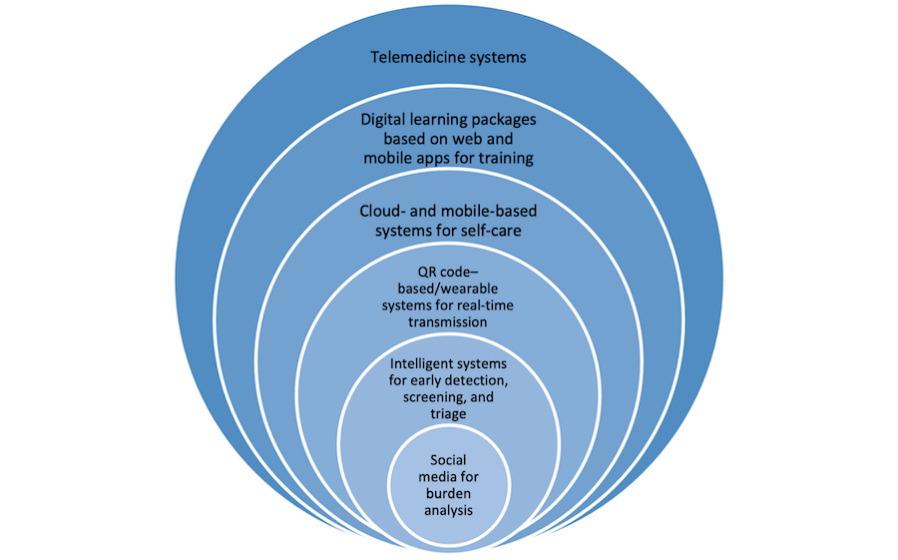
Technologies currently being applied to address the COVID-19 pandemic. QR: quick response.

Telemedicine systems are highly used in many countries. In European countries, tracking of patients was adopted due to its feasibility in smaller countries; also, home care and self-care receive a relatively large amount of focus in these countries. Intelligent systems, CDSSs, and intelligent triage systems are not well adopted due to the need to supply them with data. These data are being gathered worldwide. Furthermore, analysis of social health data could be interesting, although little research has been done in this regard. Figure 1 shows the extent of the technologies developed for fighting the COVID-19 pandemic in the literature.

Overall, in the studied countries, after the alarm was raised regarding the pandemic, implementation of eHealth strategies began immediately. mHealth solutions and large-scale deployment of virtual consultations were launched. Data analysis approaches are being applied to support decision makers, and websites and electronic training tools are being used to improve patients’ protective behaviors. Although this study presents digital tools that are being applied for pandemic control in general, it lacks evaluation of the exact outcomes of using these digital health tools; thus, further studies are needed to evaluate the effects and outcomes of using digital health tools. This study could help health policy makers make decisions regarding the investment of these tools to control COVID-19.

### Conclusion

This study reviewed the digital health tools to fight COVID-19 that have been reported in the 10 countries in which the disease is most prevalent. Although there is no equal strategy to apply digital health tools across the affected countries for pandemic control, these tools are among the primary policies that governmental and private companies have considered for disease control. The United States has developed the most technologies to fight the pandemic. Furthermore, China, the first country that was affected by COVID-19, has applied a great number of digital tools, such as epidemiological indicators, analysis platforms, drones, robots, mobile apps, training websites and educational media, videoconferencing, smart infection detectors, intelligent patient tracers, and telemedicine systems. Having considered the potential of available information technologies worldwide in the 21st century, particularly in developed countries, it appears that more digital health products, especially intelligent products, remain to be created and applied for the management of viral infections and other health crises.

## Abbreviations

CDSS: clinical decision support system
GIS: geographic information system
mHealth: mobile health
NHS: National Health Service
QR: quick response
